# Transcatheter arterial embolization followed by surgical laparotomy for hemorrhagic shock due to intestinal bleeding: a case report

**DOI:** 10.1186/s40792-022-01363-3

**Published:** 2022-01-17

**Authors:** Sayumi Kurita, Kazuo Kitagawa, Naoki Toya, Mutsumi Kaji, Satoshi Yoshioka, Yuki Hiramoto, Shuichi Fujioka, Naoto Takahashi, Ken Eto

**Affiliations:** 1 Department of Surgery, The Jikei University Kashiwa Hospital, 163-1 Kashiwashita, Kashiwa, Chiba 277-8567 Japan; 2 Department of Surgery, The Jikei University School of Medicine, 3-19-18 Nishi-shinbashi, Minato-ku, Tokyo, 105-8471 Japan

**Keywords:** Intestinal bleeding, Jejunal diverticula, Hemorrhagic shock, Intestinal amyloidosis, Transcatheter arterial embolization

## Abstract

**Background:**

Acquired jejunal diverticula are relatively rare conditions. While mostly asymptomatic, they can occasionally cause life-threatening complications requiring surgical treatment. We herein report a case of hemorrhagic shock due to jejunal diverticulum with intestinal amyloidosis that was successfully managed via transcatheter arterial embolization (TAE) and surgery.

**Case presentation:**

An 80-year-old female presenting with hematochezia and hemorrhagic shock was transferred to our institution. Contrast-enhanced computed tomography revealed extravasation in the small bowel around the upper jejunum. Massive transfusion was performed with subsequently planning for TAE to control bleeding followed by surgical laparotomy to evaluate the ischemic intestine. First, the second jejunal artery was selectively embolized with a 1:3 mixture of N-butyl cyanoacrylate (NBCA) and iodize oil, after which laparotomy was performed. Multiple jejunal diverticula were detected near Treitz’ ligament, and an induration of NBCA was palpable in the nearby mesentery. The intraoperative diagnosis was massive bleeding from acquired jejunal diverticula for which jejunectomy including the nearby diverticulum was performed to prevent future bleeding. Her postoperative course was stable. Histological examination of the specimen revealed several false diverticula with intestinal amyloidosis.

**Conclusion:**

Hemorrhagic shock due to jejunal diverticulum with intestinal amyloidosis is extremely rare. Combined treatment of TAE and surgical laparotomy appears to be effective, because the bleeding point can be identified by palpation of the embolic material.

## Background

While acquired jejunal diverticula are relatively rare conditions that are mostly asymptomatic, they can occasionally cause life-threatening complications requiring surgical treatment, such as diverticular hemorrhage [1, 2].

Several reports have shown that transcatheter arterial embolization (TAE) has become an established therapeutic approach for the treatment of gastrointestinal hemorrhage [3–6]. In cases with endoscopy-inaccessible bleeding point, TAE may be an effective alternative. Nonetheless, high rebleeding rates and serious complications, such as bowel ischemia, infarction, and perforation, have been reported, which are important factor associated with in-hospital mortality in some cases requiring surgical treatment or repeat embolization [5, 7–9].

We herein report a case of hemorrhagic shock due to bleeding from acquired jejunal diverticula with intestinal amyloidosis that was successfully managed through TAE and planned surgical laparotomy.

## Case presentation

An 80-year-old female presenting with hematochezia and hemorrhagic shock was transferred from another hospital to our institution because of a 6-day history of melena. She had previously undergone gastrointestinal endoscopy, colonoscopy, and capsule endoscopy, none of which could identify the bleeding source. She had several medical comorbidities, including rheumatoid arthritis that required oral steroids for 10 years, steroid-induced diabetes, and colon diverticulitis, as well as surgical history of hip replacement, knee replacement, cholecystectomy, and appendectomy. No relevant family and social history had been noted. No palpable mass was found on clinical examination. Her blood pressure, body temperature, heart rate, and respiratory rate were 90/63 mmHg, 36.4 °C, 117 beats per min, and 24 breaths per min, respectively.

Clinical laboratory examination showed the following data: white blood cell count of 11,300/μL, red blood cell count of 190 × 10^4^/μL, hemoglobin level of 5.6 g/dL, hematocrit of 15.6%, platelets count of 5.1 × 10^4^/μL, total protein level of 2.7 g/dL, albumin level of 1.9 g/dL, prothrombin international normalized ratio of 1.78, and activated partial thromboplastin time of 95.9 s.

**Fig. 1:**
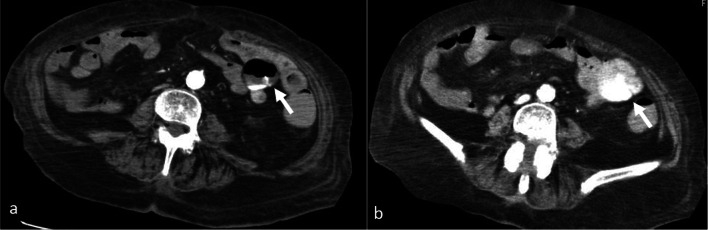
Contrast-enhanced computed tomography of the abdomen. **a** Early phase. **b** Delayed phase. Arrow: extravasation around the upper jejunum

Contrast-enhanced computed tomography (CT) of the abdomen revealed extravasation in the small bowel around the upper jejunum (Fig. 1). Massive transfusion was then performed to maintain tissue perfusion, while several treatments, including TAE or surgical repair, were performed to stop the bleeding. We planned to perform TAE to control bleeding followed by surgical laparotomy to evaluate the ischemic intestine immediately.

**Fig. 2:**
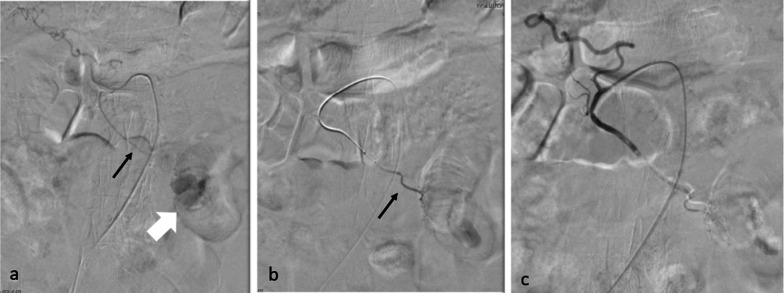
Angiography findings. **a** Second jejunal artery (black arrow) was visualized using a contrast medium. Contrast medium leakage from the peripheral artery of the second jejunal branch (white allow). **b** Second jejunal artery (black arrow) was embolized with N-butyl cyanoacrylate. **c** Leakage disappeared

First, the second jejunal artery was selectively embolized with a 1:3 mixture of N-butyl cyanoacrylate (NBCA) and iodized oil. Hemostasis was confirmed via angiography, after which the patient’s vital signs stabilized (Fig. 2).

Subsequent laparotomy revealed multiple jejunal diverticulum near Treitz’ ligament, and an induration of NBCA was palpable in the nearby mesentery. A mobile fluoroscopic X-ray system was also used to identify the NBCA location. An intraoperative diagnosis of massive bleeding from acquired jejunal diverticula was established, for which jejunectomy including the nearby diverticulum was performed to prevent future bleeding. The operative time was 164 min and the estimated intraoperative blood loss was 10 ml. Her postoperative course was stable.

**Fig. 3:**
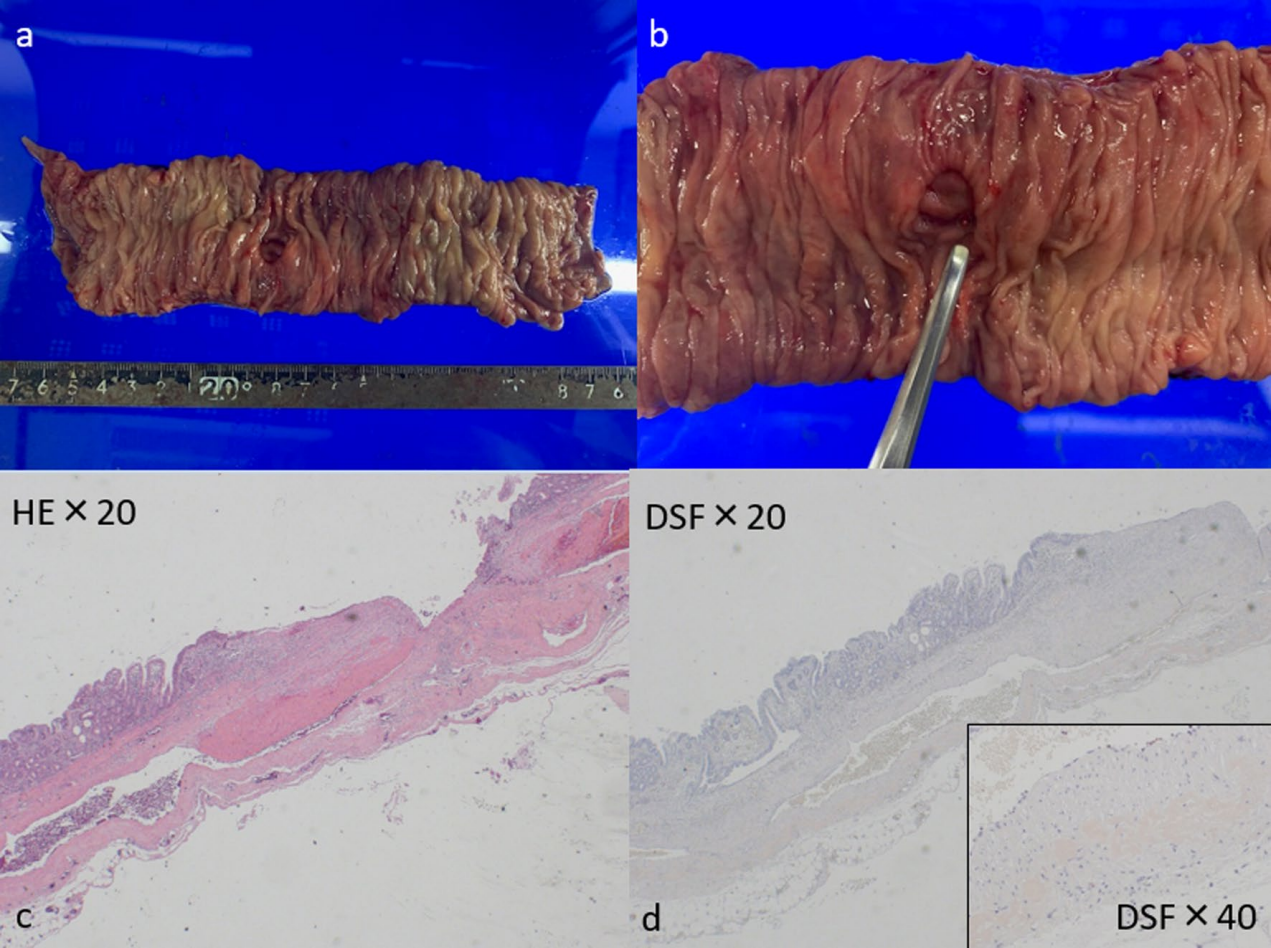
Histological examination of the specimen. **a** With macroscopic findings showed several many false diverticula. **b** Organized vein opened into the diverticula. **c** Microscopic findings revealed glass-like acidophilic unstructured deposits around the vein wall. **d** Glass-like structure was stained orange with DFS staining, which suggested intestinal amyloidosis

Histological examination of the specimen revealed several diverticula devoid of muscularis propria (false diverticula). The organized vein opened into the diverticula, which seemed responsible for the bleeding. Glass-like acidophilic unstructured deposits were found around the vein wall, which suggested intestinal amyloidosis (Fig. 3).

## Discussion

Acquired diverticula of the jejunum and ileum have a reported incidence of 0.06–2.3% [10–13]. Although jejunoileal diverticulosis is usually asymptomatic, they can occasionally cause serious complications, such as perforation or hemorrhage.

Diverticular hemorrhage remains the second most common complication of jejunoileal diverticulosis (5–33%) after diverticulitis and can cause life-threatening complications [10].

Given the extensive length and curvatures of the small intestine, examining hemorrhage is quite difficult. Gastroscopy and colonoscopy can only be used to exclude bleeding from other sites. While small bowel endoscopy has been considered a breakthrough for diagnosing intestinal disease, it remains unsuitable for hemodynamically unstable patients considering its long examination time and complexity.

Emergency surgery remains the gold standard treatment in cases with massive acute bleeding given the high incidence rates of recurrence and mortality. Yen et al. reported an overall mortality of 7.1%, while 25% of the patients whose bleeding was nonsurgically managed developed recurrence during the follow-up period. Cases without surgical resection of the bleeding lesion have shown higher mortality and recurrence rates [1].

Recently, TAE has been proven effective for the treatment of acute gastrointestinal bleeding, with high technical success and acceptable clinical success rates. Published studies have reported varying technical success rates ranging from 97 to 100% [4–8, 14–17]. The major advantage of TAE is its ability to control severe bleeding without bowel preparation. Moreover, it can be performed even in patients with unstable hemodynamics who cannot receive general anesthesia.

However, clinical success rates (percentage of patients needing no further treatment, such as a second TAE, surgery, and endoscopic treatment) have been relatively lower compared to technical success rates. Kim et al. showed that among 175 patients with lower gastrointestinal bleeding who achieved technical success, 86.1% and 6.1% achieved clinical success and developed major complications, respectively [15]. Bowel ischemia or infarction has been the most severe complication of TAE, although their published incidence rates have remained quite low [4–8, 14–17]. One study showed that cases developing bowel infarction exhibit a 90% increase in their mortality rate during hospital stay [5].

Surgical treatment should be considered in patients with brisk, ongoing bleeding. However, resection of small bowel segments must be limited give the potential risk for short bowel syndrome [18]. The complication and mortality rates following surgery for acute lower gastrointestinal bleeding have been reported to be as high as 60% and 16%, respectively [19]. Thus, careful localization of the bleeding source whenever possible prior to surgical resection is imperative to avoid rebleeding from the unresected culprit lesion [3, 19]. Andrei et al. suggested performing intraoperative endoscopy in cases, wherein the bleeding site remains unlocated, although identifying the bleeding point is generally challenging considering that intestine might be filled with blood [9].

Given that the current case was hemodynamically unstable, open surgery was initially too risky to perform. Contrast-enhanced CT showed that the bleeding site might be in the upper jejunum. As such, TAE was planned to control bleeding, after which surgical laparotomy was immediately scheduled to evaluate ischemic changes in the small intestine. Although our findings showed no sign of bowel ischemia, multiple jejunal diverticula had been detected. Considering the patient’s long-term administration of steroids, her risk of rebleeding and bowel perforation was considered to be higher than normal, which indicated jejunectomy. Intraoperative confirmation of NBCA made it easy to determine the extent of resection, and the patient was successfully managed without any fatal complications.

Moreover, histological examination of the specimen revealed intestinal amyloidosis. Although gastrointestinal bleeding is well known in patients with intestinal amyloidosis, seldom have gastrointestinal surgeries been performed in such cases [20]. Although intestinal amyloidosis could not be overlooked as the cause of the massive bleeding, our treatment strategy appeared to be appropriate and promising.

Patients whose bleeding site can be detected using contrast-enhanced CT and in whom the bleeding site is located at an endoscopically inaccessible point, such as the small intestine, are deemed suitable for undergoing this surgery. This is because achieving hemostasis by TAE will be difficult if the bleeding point cannot be identified via contrast-enhanced CT. In addition, if the bleeding point is easily accessible via endoscopy, one should consider endoscopic hemostasis first, because it is easier and less invasive than TAE or surgical approach.

Furthermore, we believe that our treatment strategy is useful regardless of the number of bleeding points. When attempting to achieve hemostasis by TAE alone in case of multiple intestinal bleeding, it may be necessary to embolize multiple or extensive arteries, increasing the risk of intestinal ischemia. However, using our strategy, surgery can be performed immediately after TAE to assess the presence of gastrointestinal ischemia and, therefore, avoid the serious complications associated with.

Nonetheless, when patients’ vital signs cannot be stabilized after TAE, our strategy might not be applicable. It is dangerous to administer general anesthesia under unstable hemodynamic conditions and may lead to the development of other underlying life-threatening conditions besides bleeding. However, when the bleeding point is detectable via angiography, but bleeding cannot be stopped due to some reason, surgery should be performed immediately to achieve hemostasis. The surgery is not also applicable in case the patient has serious comorbidity that would preclude general anesthesia, such as respiratory disorder, cardiac dysfunction, and multiple organ failure.

## Conclusion

TAE followed by surgical laparotomy seems to be an effective treatment and might be a new treatment strategy for intestinal bleeding. Prior TAE is helpful in the acute setting because of palpation of the embolic material.

## Data Availability

All data generated or analyzed during this study are included in this published article.

## Abbreviations

TAE: Transcatheter arterial embolization
CT: Computed tomography
NBCA: N-butyl cyanoacrylate
